# Which Domain of Self-Rated Health Best Predicts Medical Care Utilization Among Taiwanese Adults?

**DOI:** 10.2188/jea.JE20110144

**Published:** 2012-09-05

**Authors:** Christy Pu, Gau-Jun Tang, Yi-Ting Fang, Yiing-Jenq Chou

**Affiliations:** 1 Institute of Hospital and Health Care Administration, School of Medicine, National Yang-Ming University, Taipei, Taiwan; 2 Institute of Health and Welfare Policy, School of Medicine, National Yang-Ming University, Taipei, Taiwan; 3 Universal Eye Center, Taoyuan Branch, Taiwan; 4 Department of Public Health, School of Medicine, National Yang-Ming University, Taipei, Taiwan

**Keywords:** self-rated health, medical care utilization, adults, Short Form-36 (SF-36)

## Abstract

**Background:**

We attempted to identify the domain of self-rated health (SRH) that best predicts medical care utilization among Taiwanese adults. In addition, we examined the association between SRH and different measure of medical care utilization.

**Methods:**

We analyzed data on 11 987 community-dwelling adults aged 18 to 64 years from the 2005 Taiwan National Health Interview Survey (NHIS). NHIS data were linked to the 2006 National Health Insurance (NHI) administrative database. Then, medical care utilization in 2006, including all outpatient visits, hospitalizations, and mental health outpatient visits, was identified. Domain-specific health ratings were measured by using the Short Form-36 (SF-36) health survey questionnaire. Negative binominal models were used to estimate the contribution of the health domains to medical care utilization. Incidence rate ratios (IRRs) are presented.

**Results:**

The IRR for the physical component scale showed that those with the highest scores had 77% of the outpatient visits of those with the lowest scores. The importance of mental health domains was markedly higher in estimating mental health outpatient visits. Those with mental health scores above the median had only 61% of mental health outpatient visits of those with scores below the median.

**Conclusions:**

A person’s medical care utilization is reflected in the different domains of general health. Domain-specific measures of subjective health are not interchangeable with global general health ratings, because different domains have independent effects on medical care utilization. Our results are potentially important for medical resource allocation because they identify different health domain experiences that require improvement.

## INTRODUCTION

The use of self-rated measures of health is based on the consensus that it is important whether a person receiving medical care believes such care has achieved a desirable outcome.^1^ Self-assessed health status incorporates biological, psychological, and social dimensions of a person’s perception of their health status, which may not be accessible to an external observer and thus could be more sensitive in health monitoring.^2^ Although some studies presupposed the predictive power of self-rated health (SRH) on subsequent medical care utilization,^2^^–^^4^ it is not known if different SRH domains have different roles in such utilization. In other words, the elements of health experience that are most important in the relationship of SRH to medical care utilization remain to be determined. Such knowledge is essential for identifying potentially modifiable factors that influence utilization of medical care.

Previous research suggests that global SRH is a good risk adjustment tool in predicting mortality and medical care use.^2^^,^^3^ In the absence of laboratory and clinical indicators, SRH is a relatively inexpensive measure for identifying people that are at high risk for hospitalization.^5^ For health promotion, subjective health ratings should distinguish between a global, single-item SRH question and multidomain questionnaires, in order to determine which aspect of SRH should be targeted for improvement. In addition, analysis of how different dimensions of health experience influence medical care utilization is important in planning and providing medical services. For example, if the physical aspects of subjective health ratings tend to be more strongly associated with medical care utilization, then actions should be taken to improve the physical performance of individuals with poor subjective health ratings. However, if the mental aspects tend to have a separate effect on medical care utilization, then it is essential to improve the mental well-being of people, so as to reduce their use of medical care services.

Although there is substantial evidence that SRH is closely related to objective health measures such as subsequent mortality^6^^–^^9^ and functional decline,^10^ a person’s subjective health rating is not always consistent with his or her actual health. Moreover, studies have suggested that domain-specific health measures are not interchangeable with the single-item SRH, since certain domains of health experience are less associated with perceived overall health.^11^ One study found that different domains of health were independently associated with the global single-item SRH question, when the domain of physical functioning was more strongly associated with the global single-item SRH than were the domains of mental health and social functioning.^12^ In addition, for certain levels of perceived overall SRH, the discriminative power of different levels was weakly reflected by domain-specific measures of health.^11^ These findings suggest that research findings regarding the relationship between the single-item SRH and medical care utilization should not be generalized to domain-specific measures.

The Short Form-36 (SF-36) survey is a domain-specific health measure developed by the Medical Outcome Study (MOS) and is a useful tool for measuring subjective health. It was designed to incorporate 8 subscales: 4 assessments of physical health (physical functioning, role limitation due to poor physical health conditions, bodily pain, and general health) and 4 assessments of mental health (social functioning, vitality, role limitation due to poor emotional conditions, and mental health perceptions). The scores for the 8 subscales can also be modified to form the physical component summary (PCS) and mental component summary (MCS). The validity and reliability of the SF-36 have been extensively tested in different settings (such as patients with specific health issues),^13^^–^^15^ and different age groups,^16^^,^^17^ as well as in populations with different cultural and socioeconomic backgrounds.^18^^–^^21^ In the present study, we used the SF-36 to examine the relationship between different SRH domains and utilization of medical care among Taiwanese adults aged 18 to 64 years.

Research on medical utilization and cost often relies on self-reported data. However, such data on medical utilization and costs often suffer from recall bias and tend to be inaccurate. In studies where administrative medical care utilization data were used, the study sample often represented only a specific subgroup of the general population.^3^^,^^22^ The present research uses computerized claims records from the Taiwan National Health Insurance (NHI) database as a national representative sample.

## METHODS

### Study population

The 2005 Taiwan National Health Interview Survey (NHIS) was conducted by the Bureau of Health Promotion (BHP) in Taiwan. The subjects of that study were selected using multistate stratified systematic sampling, and the response rate was 80.6%. The target population was all individuals residing in Taiwan, as identified from the National Registry Database (sampling rate = 1.35%). The NHIS is nationally representative with proper sampling weighting. The sampling method has been described in detail in previous studies.^23^^,^^24^ The survey consisted of 15 800 individuals between the ages of 18 and 64 years (inclusive). Well-trained interviewers conducted face-to-face interviews. To identify medical expenditure and utilization for each subject, NHIS data were linked to 2006 claims data in the National Health Insurance Research Database (NHIRD), which consists of all individual medical expenditures, including both inpatient and outpatient service utilization, and their associated costs under the National Health Insurance (NHI). The NHI in Taiwan is a public insurance system with compulsory enrollment for all citizens. Almost all hospitals and clinics in Taiwan are registered in the NHI, and thus all history of medical care utilization that occurs in these institutions is recorded. All individuals enrolled in the NHIS were asked whether they agreed to have their information from the NHIS linked to their medical records in the NHIRD. Of the 15 800 subjects, 12 165 (77%) signed the consent form. Of the 12 165 subjects, 11 987 (98.5%) had complete data for the SF-36 and other variables used in this research (sex, education, and marital status) and were thus included in the present study. Data linkage was performed by the public organization in charge, and, to ensure that all individual information was protected, all individual IDs were scrambled before the dataset was released to researchers. This study was approved by the Institutional Review Board of National Yang-Ming University.

### Measures

SRH and its different domains were measured using the SF-36. The Taiwanese version of the SF-36 has been validated by previous studies.^21^^,^^25^ The raw score for each domain was transformed to a range of 0 to 100 using the standard procedure^26^; a higher score indicates better health performance. We then computed PCS and MCS scores, which were then normalized to a 100-point scale with a mean (SD) of 50 (10), based on the population of the United States. Because the NHIS is nationally representative, the PCS and MCS were normalized based on the population means and SD from the NHIS. The US factor scores were used since Taiwanese factors were not available. In addition, 1 study recommended that US factor scores be used so as to facilitate international comparisons.^27^ Medical care utilizations in 2006 were classified as number of outpatient visits, number of hospitalizations, and number of mental health outpatient visits.

### Other covariates

Covariates included in the regression models included participant baseline age, sex, educational attainment, marital status, and the Charlson Comorbidity index in 2006. The Charlson Comorbidity index contains 17 categories of comorbid conditions, which were obtained from the NHIRD using ICD-9-CM codes.^28^ A participant was defined as having a comorbid condition if the diagnose for that condition appeared at least 2 times in annual claim records.

### Statistical analysis

Table 1 shows the distribution of SF-36 subscales, medical care utilization, and other sample characteristics by age group (18–40 and 41–64 years) and sex. Medical care utilization by SRH domain is shown in Table 2. The scores for the different domains were categorized as high (scores above the median, representing better health) and low (scores below the median, representing worse health). For PCS and MCS, scores were categorized as high, medium, and low by using tertiles of the scores.

**Table 1.** Baseline sample characteristics by age and sex

			Age group					Sex				
			
	Total		18–40 years		41–64 years			Men		Women		
					
*n*	11 987		6518		5469			6242		5745		
	Mean	(s.d.)^a^	Mean	(s.d.)	Mean	(s.d.)	*P*^b^	Mean	(s.d.)	Mean	(s.d.)	*P*^b^
Physical component scale	51.2	(9.5)	53.4	(6.8)	48.5	(11.4)	<0.001	51.9	(8.9)	50.4	(10.0)	<0.001
Mental component scale	49.6	(10.2)	48.1	(10.2)	51.3	(9.9)	<0.001	50.1	(10.0)	49.0	(10.4)	<0.001
Short Form-36 subscales												
Physical functioning	95.0	(12.4)	97.3	(8.3)	92.2	(15.5)	<0.001	96.0	(11.6)	93.9	(13.1)	<0.001
Role physical	87.9	(28.9)	91.3	(24.2)	83.7	(33.3)	<0.001	89.3	(27.4)	86.3	(30.5)	<0.001
Bodily pain	84.8	(19.5)	86.0	(18.3)	83.2	(20.7)	<0.001	86.7	(18.8)	82.7	(20.0)	<0.001
General health	71.5	(20.1)	73.8	(19.0)	68.7	(21.0)	<0.001	72.8	(19.3)	70.1	(20.9)	<0.001
Vitality	68.4	(18.9)	67.9	(18.4)	69.0	(19.6)	<0.001	70.5	(18.2)	66.2	(19.5)	<0.001
Social functioning	89.5	(15.7)	89.2	(15.2)	89.8	(16.4)	<0.001	90.0	(15.6)	88.9	(16.0)	<0.001
Role emotional	83	(33.5)	80.8	(34.3)	84.5	(32.5)	0.04	83.0	(33.3)	82.0	(33.8)	0.12
Mental health	75	(16.8)	73.4	(16.3)	76.3	(17.2)	<0.001	76.2	(16.3)	73.1	(17.1)	
Number of outpatient ​ visits in 2006	13.0	(14.8)	10.0	(12.0)	16.5	(16.9)	<0.001	10.8	(13.6)	15.4	(15.7)	
Number of hospitalizations ​ in 2006	0.09	(0.4)	0.08	(0.4)	0.11	(0.5)	<0.001	0.09	(0.5)	0.10	(0.4)	0.25
Number of mental health ​ outpatient visits	0.55	(3.2)	0.34	(3.3)	0.80	(3.2)	<0.001	0.47	(2.7)	0.63	(3.7)	<0.001
Charlson Comorbidity ​ Index in 2006 (%)												
0	84.0		93.2		73.1		<0.001	83.4		84.6		0.03
1–2	11.7		5.5		19.0			11.8		11.5		
≥3	4.4		1.4		7.9			4.8		3.9		
Education (%)												
Primary school or below	16.3		1.5		34.0		<0.001	11.8		21.2		<0.001
Junior high school	15.6		11.2		20.8			17.7		13.3		
Senior high school	33.3		38.4		27.2			34.5		32.0		
University or above	34.8		48.9		17.9			35.9		33.5		
Marital status (%)												
Married/cohabitating	58.1		38.9		81.0		<0.001	56.5		59.8		<0.001
Never married	32.9		56.6		4.6			36.8		28.7		
Divorced/separated/ ​ others	9.0		4.4		14.4			6.7		11.5		

^a^s.d. = standard deviation.

^b^ANOVA (χ^2^) test for significant differences by age group and sex.

**Table 2.** All outpatient visits, hospitalizations, and mental health outpatient visits, by Short Form-36 domain

			All outpatient visits		Hospitalizations		Mental-health– related outpatient visits	
					
*n* = 11 987	*n*	Range	Mean	(s.d.)	Mean	(s.d.)	Mean	(s.d.)
Physical Component summary (PCS)^a^								
Low	3996	0–52	17.64	(17.73)	0.14	(0.59)	0.94	(3.95)
Medium	3996	52–55	11.15	(12.25)	0.07	(0.37)	0.33	(1.83)
High	3995	56–100	10.23	(12.63)	0.07	(0.39)	0.37	(3.48)
Mental Component summary (MCS)^a^								
Low	3996	0–47	13.97	(16.30)	0.11	(0.50)	0.91	(4.74)
Medium	3996	48–55	12.31	(13.88)	0.08	(0.39)	0.35	(1.98)
High	3995	56–100	12.73	(14.02)	0.09	(0.49)	0.38	(2.16)
Physical functioning^b^								
Low	3702	0–95	17.59	(17.81)	0.14	(0.59)	0.99	(4.09)
High	8285	96–100	10.96	(12.70)	0.07	(0.39)	0.35	(2.74)
Role physical								
Low	2180	0–75	18.74	(19.25)	0.18	(0.71)	1.24	(4.75)
High	9807	76–100	11.73	(13.27)	0.07	(0.38)	0.39	(2.75)
Bodily pain								
Low	5545	0–84	15.50	(16.88)	0.11	(0.53)	0.74	(4.05)
High	6442	85–100	10.86	(12.33)	0.07	(0.39)	0.38	(2.29)
General health								
Low	5889	0–72	15.38	(16.93)	0.12	(0.52)	0.80	(4.17)
High	6098	73–100	10.71	(11.94)	0.07	(0.40)	0.30	(1.89)
Vitality								
Low	5254	0–65	14.71	(16.79)	0.12	(0.51)	0.79	(4.29)
High	6733	66–100	11.68	(12.87)	0.07	(0.42)	0.36	(2.02)
Social functioning								
Low	5156	0–88	14.24	(16.53)	0.11	(0.51)	0.83	(4.36)
High	6831	89–100	12.07	(13.25)	0.08	(0.42)	0.33	(1.96)
Role emotional								
Low	2988	0–67	14.41	(17.80)	0.11	(0.50)	0.98	(5.20)
High	8999	68–100	12.54	(13.62)	0.09	(0.45)	0.40	(2.19)
Mental health								
Low	5202	0–72	14.10	(16.16)	0.10	(0.47)	0.81	(4.28)
High	6785	73–100	12.17	(13.58)	0.08	(0.46)	0.34	(2.07)

^a^Tertiles.

^b^The 8 domains were dichotomized by using score medians.

Multiple negative binominal models (to account for overdispersion of count data, which is often present in data on medical care utilization)^29^^,^^30^ were used to calculate the incidence rate ratio (IRR) for the numbers of outpatient visits, hospitalizations, and mental health outpatient visits (Tables 3 and 4).

**Table 3.** Multiple negative binomial estimation results for number of outpatient visits^a^ in 2006

	Total		18–40 years		41–64 years		Male		Female	
					
*n*	11 987		6518		5469		6242		5745	
	IRR^b^	95% CI^c^	IRR	95% CI	IRR	95% CI	IRR	95% CI	IRR	95% CI
Age, yrs										
18–40	1.00						1.00		1.00	
41–64	1.13	(1.08, 1.18)					1.26	(1.18, 1.35)	1.00	(0.94, 1.06)
Sex										
Female	1.00		1.00		1.00					
Male	0.68	(0.65, 0.70)	0.60	(0.57, 0.63)	0.78	(0.74, 0.82)				
Physical component scale (PCS)^d^										
Low	1.00		1.00		1.00		1.00		1.00	
Medium	0.83	(0.80, 0.87)	0.86	(0.81, 0.92)	0.81	(0.77, 0.86)	0.78	(0.73, 0.84)	0.87	(0.83, 0.93)
High	0.77	(0.74, 0.81)	0.78	(0.74, 0.83)	0.75	(0.70, 0.80)	0.66	(0.62, 0.71)	0.88	(0.83, 0.93)
Mental component scale (MCS)										
Low	1.00		1.00		1.00		1.00		1.00	
Medium	0.89	(0.86, 0.93)	0.88	(0.84, 0.93)	0.92	(0.86, 0.98)	0.91	(0.85, 0.97)	0.88	(0.84, 0.93)
High	0.83	(0.79, 0.87)	0.78	(0.74, 0.83)	0.87	(0.82, 0.93)	0.81	(0.75, 0.86)	0.85	(0.80, 0.90)
Education										
Primary school or below	1.00		1.00		1.00		1.00		1.00	
Junior high school	0.90	(0.84, 0.96)	0.91	(0.74, 1.12)	0.87	(0.81, 0.93)	0.88	(0.79, 0.97)	0.90	(0.83, 0.97)
Senior high school	0.92	(0.86, 0.97)	0.91	(0.75, 1.12)	0.89	(0.83, 0.95)	0.93	(0.84, 1.02)	0.86	(0.80, 0.93)
University or above	0.97	(0.91, 1.03)	0.95	(0.78, 1.16)	0.96	(0.89, 1.03)	1.01	(0.92, 1.12)	0.87	(0.80, 0.94)
Marital status										
Married/cohabitating	1.00		1.00		1.00		1.00		1.00	
Never married	0.79	(0.76, 0.83)	0.81	(0.77, 0.85)	0.75	(0.67, 0.85)	0.80	(0.75, 0.86)	0.81	(0.76, 0.86)
Divorced/separated/others	0.95	(0.90, 1.02)	0.99	(0.88, 1.11)	0.96	(0.89, 1.03)	0.86	(0.77, 0.96)	1.04	(0.97, 1.12)
Charlson Comorbidity index										
0	1.00		1.00		1.00		1.00		1.00	
1–2	2.23	(2.11, 2.36)	2.35	(2.13, 2.59)	2.17	(2.03, 2.32)	2.41	(2.22, 2.62)	2.02	(1.88, 2.17)
≥3	2.83	(2.60, 3.08)	2.87	(2.36, 3.50)	2.74	(2.49, 3.02)	3.00	(2.66, 3.38)	2.46	(2.18, 2.78)

^a^All outpatient visits.

^b^IRR = incidence rate ratio.

^c^CI = confidence interval.

^d^Tertiles.

**Table 4.** Multiple negative binomial estimation results for Short Form-36 (SF-36) health domains^a^ and medical care utilization in 2006^b^

	Number of outpatient visits		Hospitalizations		All mental-health–related outpatient visits	
			
*n* = 11 987	IRR^c^	95% CI^d^	IRR	95% CI	IRR	95% CI
Age, yrs						
18–40	1.00		1.00		1.00	
41–64	1.13	(1.08, 1.18)	0.50	(0.40, 0.61)	1.50	(1.19, 1.88)
Sex						
Female	1.00		1.00		1.00	
Male	0.68	(0.66, 0.71)	0.83	(0.71, 0.98)	0.84	(0.69, 1.01)
SF-36 subscales						
Physical functioning	
Low	1.00		1.00		1.00	
High	0.95	(0.91, 0.99)	0.78	(0.65, 0.94)	0.79	(0.63, 1.00)
Role physical						
Low	1.00		1.00		1.00	
High	0.87	(0.83, 0.92)	0.77	(0.62, 0.95)	0.59	(0.45, 0.79)
Bodily pain						
Low	1.00		1.00		1.00	
High	0.87	(0.83, 0.90)	0.90	(0.76, 1.07)	1.19	(0.96, 1.47)
General health						
Low	1.00		1.00		1.00	
High	0.89	(0.86, 0.93)	1.03	(0.86, 1.24)	0.75	(0.60, 0.94)
Vitality						
Low	1.00		1.00		1.00	
High	0.99	(0.95, 1.03)	0.80	(0.66, 0.96)	1.39	(1.09, 1.77)
Social functioning	
Low	1.00		1.00		1.00	
High	1.00	(0.96, 1.04)	0.91	(0.76, 1.09)	0.59	(0.47, 0.75)
Role emotional						
Low	1.00		1.00		1.00	
High	1.02	(0.97, 1.06)	0.99	(0.81, 1.20)	0.82	(0.64, 1.05)
Mental health						
Low	1.00		1.00		1.00	
High	0.96	(0.92, 1.00)	1.10	(0.91, 1.33)	0.61	(0.48, 0.77)

^a^All models controlled for education, marital status, and Charlson Comorbidity Index.

^b^The 8 domains were dichotomized by using score medians.

^c^IRR = incidence rate ratio.

^d^CI = confidence interval.

The initial regression analysis used only the PCS and MCS for outpatient visits (Table 3). Because the purpose of this study was to determine how the SF-36 and its domains are related to medical care utilization, we estimated the regression by using the 8 domains instead of the PCS and MCS (Table 4). We also subdivided mental health outpatient visits, based on the International Classification of Diseases, 9th Revision (ICD-9). The NHI claim system allows physicians to enter 3 diagnoses for each outpatient visit. A mental health outpatient visit was defined as a consultation in which a patient received an ICD-9 diagnosis beginning with 290–319 for any of the 3 diagnoses assigned for that outpatient visit.

Statistical significance was calculated based on the weighted sample. The sampling weight was provided by the BHP. Because the 8 SF-36 domains could be interdependent, collinearity was a concern. However, Pearson correlation coefficients showed that only 1 of 28 correlations between domains exceeded 0.6 (vitality and mental health, 0.69); hence, collinearity should not be a major concern in this study. A similar method and cutoff point for checking collinearity of domains of subjective health were used by Kempen et al.^11^ The statistical software package STATA MP/10.1 (Stata Corp, LP College Station, TX, USA) was used.

## RESULTS

Table 1 shows the distribution of SF-36 scores and other variables by age group and sex. As compared with the older age group (41–64 years), the younger age group (18–40 years) had higher PCS and PCS scores and physical performance; however, the older age group had slightly higher scores for the mental health domains. The younger age group was more highly educated and more likely to be single as compared with the older group. Scores for most domains were higher among men than among women.

Table 2 shows total outpatient visits, hospitalizations, and mental health outpatient visits by SF-36 domain. For all health domains, including PCS and MSC, patients with scores below the median had higher average utilization for all 3 measures of medical care, and these results were similar between age groups and sexes.

Regarding number of outpatient visits (Table 3), those with higher PCS and MCS scores had lower IRRs. For example, among the total sample (*n* = 11 987), those in the group with the highest PCS scores (representing best physical health) had only 0.77 times the outpatient visits of those in the group with the lowest scores. The results did not differ by age group or sex.

The relationships between the different SRH domains and the 3 types of medical care use are shown in Table 4. The physical health domains (physical functioning, role physical, bodily pain and general health) were significant determinants of number of outpatient visits. Physical functioning, role physical, and vitality were significantly associated with subsequent hospitalization. Regarding mental health outpatient visits, the mental-health domains were more important compared with that for general outpatient visits and hospitalizations. For example, individuals with scores above the median for social functioning and mental health had significantly lower IRRs for mental health outpatient visits, and the IRRs were much lower than the IRR for all outpatient visits. Vitality also became significant (IRR = 1.39, 95% CI = 1.09–1.77).

Table 4 shows that the older age group was less likely to be hospitalized (IRR = 0.50). The IRR for the older age group was 1.24 (95% CI = 0.26–1.46) in univariate analysis (not shown) but decreased to less than unity when other variables (sex, education, marital status, and Charlson Comorbidity Index) were added.

## DISCUSSION

Although previous research suggested that global SRH was a predictor of medical care utilization, those studies identified neither the elements of this subjective measure that were most important with regard to medical care utilization nor the types of medical utilization that were more strongly associated with SRH. We found that domains of health ratings had varying effects on utilization of medical care. Future studies need to determine exactly what each subscale consists of and what the mechanisms are for the association, as such analysis is beyond the scope of this study. Our findings could be important for future research that uses subjective health as a predictor of medical care utilization, as well as for planning and providing medical services.

Our data allowed us to analyze a general population of adults aged 18 to 64, using a nationally representative sample with a sufficient sample size. Unlike survey data, which tend to suffer from participant recall bias and unwillingness to report, the computerized data on medical claims provided us with accurate measures of medical care utilization.

This study had some limitations. First, the results do not allow us to infer a cause–effect relationship between SRH domains and utilization of medical care. Second, the SF-36 is only 1 form of subjective health rating; thus, the SF-36 domains might not encompass all aspects of subjective health ratings. For example, 1 study found that age, early-life factors, family history, sociodemographic variables, psychosocial factors, health behavior, and health (such as sickness absences) together explained less than 45% of the variance in global SRH for men and women.^31^

While physical functioning is frequently found to be more closely associated than mental function with subjective health ratings and objective health measures such as mortality, our results show that mental function should not be overlooked, because mental health medical utilization was more closely associated with mental health assessment. Thus, in explaining medical care utilization, SRH should be separated into different domains on the basis of the type of medical care being examined.

The importance of our findings lies in their practical implications. Previous studies have shown that global SRH is a good predictor of mortality and use of medical care. Our results add to these findings by identifying, in a specific manner, those who are at risk of high medical care use. This will make interventions more practical by allowing development of interventions that target people with specific poor domain experiences. For example, we found that among the physical domains, bodily pain had a marked effect on outpatient visits: those with higher scores (ie, less pain) had a significantly lower IRR for number of outpatient visits. The IRRs were much lower than those for physical functioning, which suggests that people visit outpatient departments when they experience pain, and although pain might be associated with actual physical health, its effect might not be explained by other physical health domains. This is consistent with previous studies, which found that pain is often related to medical care utilization.^32^ Pain management is thus important in reducing medical care utilization.

Our results show that social functioning is a significant determinant of mental health outpatient visits. Previous studies also suggested that loss of social functioning often explains the occurrence of mental illnesses, such as depression,^33^^,^^34^ that may require medical care utilization. An interesting finding is that those with higher vitality scores were more likely to have a higher number of mental health outpatient visits, possibly because the existence of a mental health problem is not solely reflected by vitality. Alternatively, people with higher vitality may be more likely to seek help when they have a mental health problems. It is also possible that there are interactive effects among the health domains. However, testing for interactive effects between health domains was beyond the scope of this study. Future studies should also investigate the reasons for the poor self-health ratings in different domains.

In a comparison with studies done in other countries, the distribution of scores for the different domains in our sample was similar to that of Mavaddat et al^12^ (20 853 UK adults), though the mean score in our sample was higher. Vitality and general health had the lowest mean scores in both samples. The mean scores for the domains in our sample were also higher than those from Nordlund et al^35^ (9489 Swedish adults), which shows that our sample was (subjectively) healthier. Whether our results can be generalized to other countries should be further investigated.

Our results showed that the physical and mental aspects of SRH independently influence medical care utilization, apart from the Charlson Comorbidity Index. This indicates that people sometimes include factors other than the presence of health conditions in rating their health. Previous studies have shown that individuals with the same disease rate their health differently due to factors other than their disease status. For example, Thomas et al^36^ found that, among people with type 2 diabetes mellitus and coronary artery disease, those who had regular employment and exercised regularly had significantly higher self-health ratings than did retired or unemployed individuals who did not exercise regularly. Similarly, Tsai et al^37^ found that healthy behaviors are associated with an increased likelihood of reporting optimal SRH among adults with cardiovascular diseases or diabetes. These findings suggest that aspects of a person’s subjective health rating can change regardless of disease status. For example, given the same level of objective health and ability to function, one person might feel less need than another person to reduce daily activities (as measured by the role physical domain) because, for example, he or she has better health behaviors or more family support.

Domain-specific measures of subjective health are not interchangeable with global general health ratings, because different domains have varying, independent effects on medical care utilization. The different subscales in the physical and mental health domains contribute differently to medical care use. To reduce medical care utilization, improvements in specific health domain experiences should be targeted.
